# Recent Advances in Heavy Metal Stabilization and Resource Recovery from Municipal Solid Waste Incineration Fly Ash

**DOI:** 10.3390/toxics13080695

**Published:** 2025-08-20

**Authors:** Yunfei He, Yue Jiang, Lingwei Ren, Chenyiyi Qian, Han Zhang, Yuchi Zhong, Xuetong Qu, Jibo Dou, Shuai Zhang, Jiafeng Ding, Hangjun Zhang

**Affiliations:** 1 School of Engineering, Hangzhou Normal University, Hangzhou 310018, China; heyyff@hznu.edu.cn (Y.H.); 2024111032003@stu.hznu.edu.cn (Y.J.); 2024211102020@stu.hznu.edu.cn (C.Q.); 2022210308076@stu.hznu.edu.cn (H.Z.); qxt912068192@163.com (X.Q.); 20240074@hznu.edu.cn (J.D.); zs@hznu.edu.cn (S.Z.); zhanghangjun@hznu.edu.cn (H.Z.); 2 Zhejiang Provincial Key Laboratory of Wetland Intelligent Monitoring and Ecological Restoration, Hangzhou 311121, China; 3 School of the Environment, Nanjing University, Nanjing 210023, China; 4 Hangzhou Fuyang Huilong Environmental Protection Technology Co., Ltd., Hangzhou 330183, China; mmc6767@zju.edu.cn (L.R.); sll20200103@163.com (Y.Z.)

**Keywords:** municipal solid waste incineration fly ash, heavy metal, removal, recovery

## Abstract

Municipal solid waste incineration fly ash (MSWI FA) is recognized as a hazardous solid waste due to its enrichment in toxic heavy metals and high leaching potential. This review systematically summarizes the current understanding of heavy metal occurrence in MSWI FA and associated environmental risks. Solidification and stabilization methods, such as cement-based curing and chemical immobilization, are widely applied due to their cost-effectiveness and operability, though their long-term stability and recovery potential remain limited. Thermal treatment technologies, including sintering, vitrification, thermal separation, and molten salt processes, have shown excellent performance in reducing volume and enhancing the immobilization or recovery of heavy metals. However, these methods are often limited by high energy demands and operational complexity. Recently, emerging technologies such as electrodialysis, bioleaching, and electrokinetic remediation have demonstrated promising capabilities for selective metal recovery under relatively mild conditions. Nevertheless, these novel approaches remain at an early stage of development and have thus far been validated only at the laboratory or pilot scale. Overall, integrating multiple treatment technologies while advancing resource-oriented and low-carbon approaches will be essential for the sustainable management of MSWI FA.

## 1. Introduction

**Figure 1:**
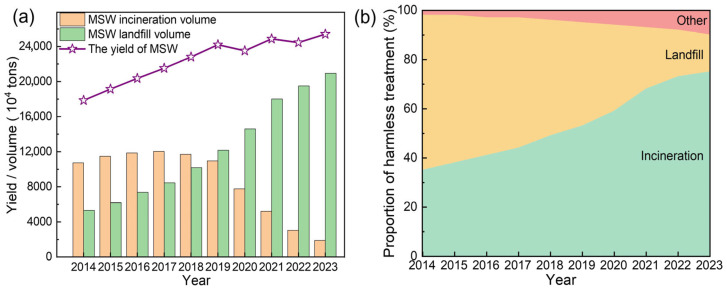
Trends in the total volume and treatment of MSW in China from 2014 to 2023. (**a**) Changes in the yield, incineration, and landfilling of MSW in China. (**b**) Proportions of MSW undergoing harmless treatment (including incineration, landfilling, and other methods).

According to the World Bank’s What a Waste 2.0 report, global municipal solid waste (MSW) generation reached approximately 2.01 billion tons in 2016 and is projected to exceed 3.4 billion tons by 2050 under a business-as-usual scenario [1]. The generation of MSW in China also has shown a continuous upward trend [2]. According to the National Bureau of Statistics of China (NBSC) [3,4,5,6,7,8,9,10,11,12], the total MSW volume increased from 179 million tons in 2014 to 254 million tons in 2023, reflecting an average annual growth rate of 4.2%, which was illustrated in Figure 1a. A notable exception occurred between 2019 (242 million tons) and 2020 (235 million tons), where a temporary decline was observed-likely influenced by disruptions associated with the COVID-19 pandemic. However, MSW generation rebounded post-2021, culminating in a 42.2% increase by 2023 compared to 2014. Concurrently, China’s waste management landscape has undergone a profound structural change (Figure 1b), achieving near-complete safe disposal of MSW. In 2014, landfill treatment dominated the waste disposal sector, accounting for 63% (107 million tons) of processed waste. However, by 2023, this share had dropped drastically to 15% (18.9 million tons). In contrast, waste-to-energy incineration had emerged as the primary treatment pathway, with its proportion soaring from 35% (53.3 million tons) in 2014 to 75% (210 million tons) in 2023. Meanwhile, the adoption of alternative waste treatment technologies (other, e.g., composting, recycling) had also expanded, with their collective share increasing from 2% to 10% over the same period (Table S1).

**Figure 2:**
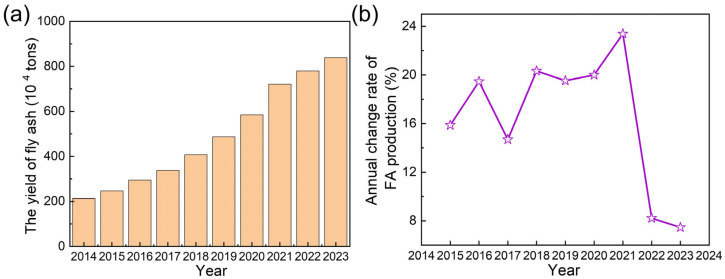
(**a**) The yield of MSWI FA and (**b**) annual change rate of FA production from 2014 to 2023.

China’s National Plan for the Construction of Municipal Solid Waste (MSW) Harmless Treatment Facilities set a target for incineration to exceed 50% of total waste disposal by 2020. In practice, the incineration efficiency surpassed expectations, reaching 59% in 2020 and further rising to 75% by 2023 (Figure 1b). This corresponded to an incineration capacity of 862,000 tons per day, representing a staggering 363% increase from 186,000 tons per day in 2014 [13]. Incineration technology offered significant waste reduction benefits with high volume and mass reduction [14,15,16]. However, it also generated fly ash (FA), a byproduct that presented environmental management challenges [17]. Based on an estimated 4% generation rate relative to MSW incineration, annual FA production rose from 2.1 million tons in 2014 to 8.4 million tons in 2023, exhibiting a 293% cumulative increase over the decade (Figure 2a). The growth rate of FA production exhibited dynamic fluctuations. From 2014 onward, annual FA output increased gradually, reaching a peak growth rate of 23.4% in 2021. Then, the growth rate increase slowed, with the annual growth rate moderating to 7.5–8.2% in 2022 and 2023 (Figure 2b).

MSWI FA posed a severe environmental and public health threat, as it not only contained persistent organic pollutants (POPs) but also served as a sink for highly toxic heavy metals [18,19,20,21]. These metals exhibited high environmental mobility, worsening their potential ecological risks. Under acid rain conditions, the leaching concentrations of Pb and Cd from FA exceeded hazardous waste leaching toxicity thresholds by several orders of magnitude [22]. Once released into the ecosystem, these heavy metals migrated through rainwater leaching, atmospheric deposition, and biological accumulation, ultimately entering the food chain.

Given these concerns, the removal and stabilization of heavy metals in FA has become a critical challenge in MSWI waste management. This review aims to systematically analyze the pollution characteristics and environmental impacts of heavy metals in fly ash, and to evaluate major treatment technologies, including solidification/stabilization, thermal treatment (sintering, vitrification, and thermal separation), and recovery-oriented strategies such as chemical leaching and electrochemical separation. These methods are discussed based on their principles, advantages, limitations, and recent innovations. By integrating insights from both conventional and emerging approaches, this review provides a foundation for developing resource-oriented and low-carbon solutions to support the sustainable management and valorization of MSWI FA.

## 2. Occurrence and Migration Behavior of Heavy Metals in FA

### 2.1. Sources and Composition of Heavy Metals in FA

MSWI FA was a fine powder collected by the flue gas cleaning system during the incineration of household waste. It is typically grayish-white or dark gray [23,24]. FA was highly hygroscopic and easily airborne, with particle sizes mainly below 100 μm [15,25,26,27]. The particles had irregular shapes, including spherical, flaky, and fibrous forms [28,29,30,31,32]. FA had an amorphous structure and a large specific surface area (5–950 m^2^/kg) [19,33,34,35,36], which gives it high surface reactivity. These properties promoted the adsorption and migration of pollutants. Its chemical composition was mainly based on the CaO-SiO_2_-Al_2_O_3_ system [37,38]. However, the exact composition varied by region and season due to differences in feedstock, incineration conditions, and gas treatment systems [32]. FA also contains a variety of soluble salts, such as NaCl, KCl, and Na_2_SO_4_, which are primarily derived from the Cl^−^ and SO_4_^2−^ in plastics and food waste [39], primarily derived from the combustion of plastics and the salts in food waste. The presence of salts significantly increased the leaching risk of heavy metals, primarily due to complexation reactions between metal ions (e.g., Pb^2+^, Cd^2+^) and anions (e.g., Cl^−^, SO_4_^2−^) [40,41]. These complexes enhanced the solubility and mobility of heavy metals in aqueous environments, thereby promoting their release. In addition, common elements like Ca, K, S, Si, Mg, Fe, Al, and C are typically found in mineral forms such as Ca(OH)_2_, CaSO_4_, CaCO_3_, KCl, and NaCl [15,17,23,42,43,44,45].

### 2.2. Distribution of Heavy Metal in FA

**Figure 3:**
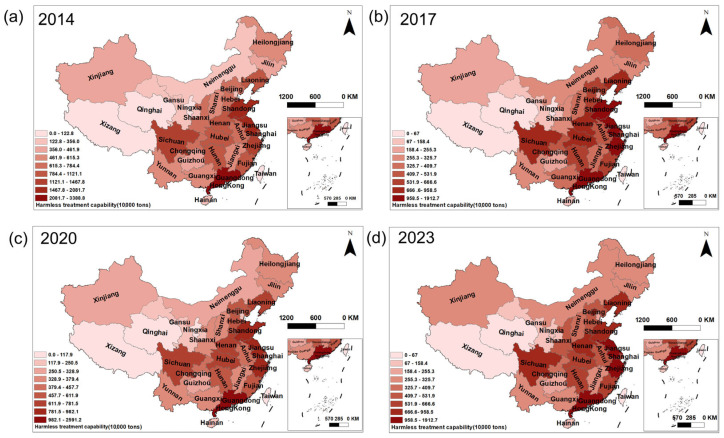
MSW collection volumes across provinces in China. (**a**) 2014, (**b**) 2017, (**c**) 2020, and (**d**) 2023.

The volume of MSW treated in China had shown a continuous increase from 2014 to 2023 (Table S2), accompanied by distinct regional disparities (Figure 3). The generation of MSW was predominantly concentrated in the economically developed eastern and coastal provinces, such as Zhejiang, Jiangsu, Shandong, and Guangdong. In the central region, provinces like Sichuan also reported relatively high MSW generation.

The classification of MSWI FA as hazardous or non-hazardous is determined by national and international regulations based on its leaching characteristics and pollutant content. In the European Union, it is typically classified as hazardous waste under the European Waste Catalogue (EWC) due to the presence of leachable heavy metals and persistent organic pollutants like dioxins. This classification relies on tests such as EN 12457 and the threshold values defined in the EU Landfill Directive (1999/31/EC) and Council Decision 2003/33/EC. In China, the GB 18598-2019 standard sets lower leaching limits for key metals. Fly ash that exceeds these thresholds must be treated, typically through stabilization or solidification, prior to landfill disposal.

**Figure 4:**
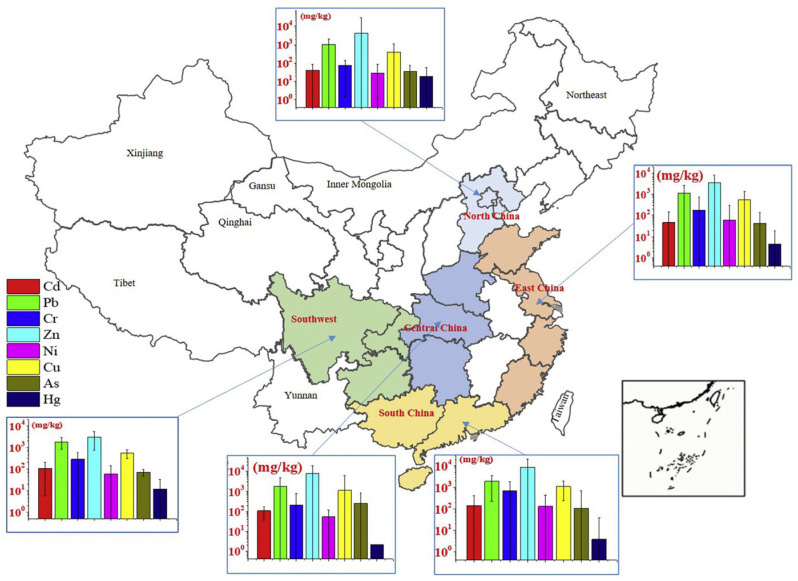
Geometric mean values of heavy metal concentrations in MSWI FA from different regions of China, reproduced from [46]. 2019, Environ Pollut.

The types and concentrations of heavy metals in FA varied across different regions, as shown in Figure 4 [46]. In southern China, FA from MSWI typically contained higher levels of Cd, Pb, Cr, Zn, and Ni [13]. The Cd, Pb, and Cr in urban waste mainly come from paper and plastics. In economically developed southern regions, the amount of paper and plastics in urban waste was higher than in other areas, which explained why the FA in these regions had elevated levels of Cd, Pb, Cr, Zn, and Ni. In central regions, As and Cu concentrations tended to be higher. In northern regions, the highest levels of Hg were detected in FA from municipal waste incinerators, likely due to the widespread use of coal for cooking and heating during winter, which increased the proportion of coal ash in the waste stream. Similarly, in the southwestern region, relatively high Cd concentrations in FA may be attributed to the incineration of locally mined coal rich in Cd or the co-incineration of coal ash mixed into municipal solid waste [47].

The distribution of heavy metals in MSWI FA was mainly influenced by thermal volatilization behavior and regional differences. At typical incineration temperatures (800–1000 °C), metals undergo processes like thermal decomposition, volatilization, and condensation, leading to different patterns of accumulation [32,48]. Generally, low-volatility metals like Cr, Cu, Mn, and Ni tended to stay in the bottom ash, mainly in the form of stable oxides or silicates [49]. Semi-volatile metals, such as As, Sn, Zn, Pb, and Cd, were more likely to condense on the surface of FA particles as the flue gas cools [50]. Highly volatile metals, like Hg, mostly escaped with the gas phase, and only a small portion was captured in the ash. As a result, the concentrations of Zn, Pb, and Cd in FA were often 5 to 20 times higher than those found in bottom ash [51].

### 2.3. Environmental Risks of Heavy Metals in FA

The environmental risk of MSWI FA mainly came from the active chemical forms of heavy metals and their potential to migrate across multiple environmental media. Speciation analysis showed that about 20–30% of Cd and Pb in FA existed in the acid-extractable and oxidizable fractions [18]. These forms could be quickly released under weakly acidic conditions (pH 4–6) [32,52]. Although Zn reached a total concentration of up to 9100 mg/kg, much of it was bound in stable silicate forms. As a result, its leaching concentration remained low, at only 5.22 mg/L. The leaching concentrations of Pb and Cd could greatly exceed national landfill limits (0.25 mg/L for Pb and 0.15 mg/L for Cd), reaching up to 2.51 mg/L and 0.37 mg/L, respectively [53], which represented a significant leachate pollution risk [32]. Moreover, heavy metals in FA often co-existed with high levels of chlorides (15–25 wt.% Cl) and dioxins (0.5–50 ng TEQ/g), forming complex pollutant systems. Dioxins are highly toxic and persistent organic pollutants that pose serious risks to both the environment and human health. Their formation in MSWI FA is closely associated with the presence of chloride ions and high-temperature combustion processes. Depending on the treatment method, dioxins may be destroyed, released, or immobilized [54]. Even when MSWI fly ash undergoes proper stabilization and is disposed of in engineered landfills, residual environmental risks remain [55]. The primary concerns are the potential leaching of toxic heavy metals (e.g., Pb, Cd, Zn, Cr, Ni) and persistent organic pollutants (e.g., dioxins). Under long-term water-rock interactions in landfills, these pollutants could migrate through various pathways such as leachate transport, groundwater diffusion, and plant uptake, eventually entering the ecological food chain [56]. Due to the bioaccumulation and biomagnification effects of heavy metals, they could be transferred through the food chain to humans. This may lead to serious health effects, including neurotoxicity, kidney damage, and carcinogenicity [57,58]. Therefore, it was essential to properly manage and treat heavy metals in MSWI FA to reduce their environmental and health risks.

## 3. Non-Recycling Pathways: Immobilization and Stabilization Technologies

Solidification/stabilization involved the addition of chemical agents to MSWI FA to immobilize hazardous components through physical encapsulation or chemical transformation. Widely employed for the treatment of heavy metals in FA, solidification/stabilization was generally classified into two core techniques: solidification and stabilization [55].

### 3.1. Solidification Technology

**Figure 5:**
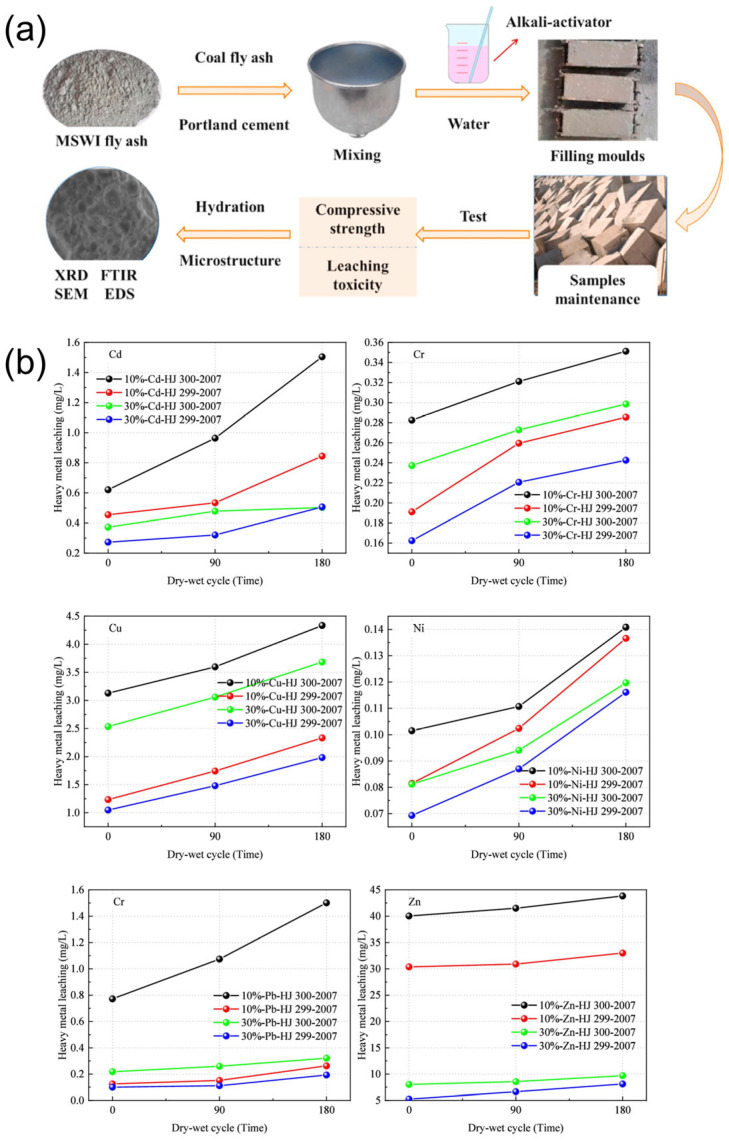
(**a**) Mechanism of stabilization of MSWI FA [61]. 2021, J. Clean. Prod. (**b**) Heavy metal leaching of incineration FA, reproduced from [64]. 2025, Langmuir.

Solidification typically entailed the incorporation of binding materials—such as cement [33,59], clay [60], or other mineral additives—into FA to physically entrap or chemically bind heavy metal ions, thereby reducing their mobility and leachability (Figure 5a) [61]. This approach effectively reduced their leaching toxicity by forming a stabilized matrix through physical encapsulation, enhancing long-term environmental safety. However, this process typically resulted in an increase in the volume of the treated product, with approximately 300–400 kg of cement and water required per ton of FA for successful immobilization [62]. The hydration reactions generated new matrices that encapsulate the FA, increasing the mass and volume by up to 40%, which caused a higher burden on landfill space [63]. It was worth noting that the cement dosage and exposure duration played a crucial role in the leaching behavior of heavy metals [64]. When incineration FA mixed with 10% cement underwent 180 dry-wet cycles, Cd leaching exceeded 40.75% of the threshold set by the Chinese standard GB 18598-2019. In contrast, samples with a 30% cement content remained within regulatory limits, with Cd leaching reaching 84.45% of the standard. Moreover, after 180 dry-wet cycles, the leaching concentration of heavy metals increased by an order of magnitude compared to the initial levels (Figure 5b), highlighting the potential long-term environmental risks associated with this process.

### 3.2. Stabilization Technology

Stabilization involved the use of additives to convert pollutants in MSWI FA into substances with lower toxicity, reduced mobility, and poor solubility, minimizing the risk of pollutant leaching [32]. Chemical stabilization, in particular, reduced environmental risks by introducing reagents that transformed heavy metals into less soluble or less toxic forms. This method offered several advantages, including high efficiency, simplicity, and minimal volume increase post-treatment, making it an effective option for harmless waste management and heavy metal immobilization [32,65]. Common chemical stabilization techniques included precipitation-dissolution and composite precipitation methods. The precipitation-dissolution process stabilized heavy metals by forming low-solubility precipitates when specific reagents were added. These reagents, such as inorganic or organic components, converted heavy metals into insoluble inorganic minerals, reducing their leaching potential [32].

Phosphate-based stabilization had been widely recognized as an effective approach to reduce the leaching of heavy metals from MSWI FA [45]. Experimental results had shown that phosphate-treated samples exhibited a significantly lower Zn leaching concentration (5.89 mg/L) compared to raw FA (9.24 mg/L), indicating enhanced immobilization under low pH conditions [66]. The formation of stable phosphate minerals, such as Cd_3_(PO_4_)_2_ and Zn_3_(PO_4_)_2_, also contributed to the reduced mobility of Cd and Cr, further confirming the efficacy of phosphate-induced immobilization. However, chemically stabilized FA still posed a potential risk of heavy metal leaching under acidic conditions. Meanwhile, phosphorus is a finite resource from phosphate rock, and it is also essential for food production as a key component of fertilizers. Therefore, while phosphate-based stabilization offers significant environmental benefits, its long-term sustainability and the availability of phosphorus must be considered.

Ligands, such as oligomeric dithiocarbamate (ODTC), have been employed to enhance the stabilization of heavy metals in laboratory-scale tests [36]. This approach achieved over 95% reduction in the leaching of Pb, Cd, and Cu. At a FA incorporation rate of 20%, all measured leaching concentrations remained below regulatory limits, indicating effective long-term stabilization. X-ray diffraction (XRD) and speciation analyses confirmed the formation of stable chemical bonding between the ODTC ligands and target metal ions, demonstrating improved acid resistance and immobilization efficiency.

Composite precipitation technologies leveraged organic chelating agents to form stable coordination complexes with heavy metals, thereby reducing their mobility and environmental risk [32]. Common organic reagents include dithiocarbamates (DTC) and thiourea derivatives [67]. The DTC and thiourea were investigated as chelating agents for stabilization effects on Pb, Cd, and Ni in MSWI FA [35]. In particular, DTC addition at 1% (*w*/*w*) reduced Pb and Cd leaching to 0.046 mg/L and 0.21 mg/L, respectively, outperforming thiourea, which showed less effective stabilization and exceeded allowable limits for some metals. These findings highlight the superior chelating performance of DTC, especially for Pb and Cd.

However, the use of organic chelating agents alone is limited by factors such as high cost, difficulty in procurement, and potential ecological toxicity [27]. Consequently, the development of composite chemical agents combining inorganic chemicals and organic chelating agents has become a key research focus. For instance, phosphate-based treatment significantly reduced Zn leaching, while chelating agents further immobilized Pb by forming insoluble phases such as Pb_3_(PO_4_)_2_ and Pb_5_(PO_4_)_3_OH [66]. The synergistic use of ternary mixtures, such as trithiocyanuric acid trisodium salt (TMT), sodium dihydrogen phosphate, and sodium dimethyldithiocarbamate (TMT-NaH_2_PO_4_-SDD), had been shown to enhance heavy metal stabilization, reducing Pb and Cd leaching concentrations to as low as 0.035 mg/L and 0.002 mg/L, respectively [68].

Despite the operational simplicity and effectiveness of solidification/stabilization (S/S) technologies, several technical limitations remain. Cement-based binders, for instance, tend to increase the mass and volume of treated residues, thereby elevating transport and disposal costs. Additionally, high FA content can compromise the mechanical strength of the final product, limiting its suitability for landfill disposal [59]. To overcome these challenges, an integrated approach was proposed that incorporates supplementary cementitious materials (e.g., silica fume, blast furnace slag, and coal FA) alongside eco-friendly stabilizers such as potassium dihydrogen phosphate and wood-derived biochar [69]. This method enhanced the formation of calcium–silicate–hydrate (C–S–H) gels and reduced Pb leachability by 36.3% at a 20% silica fume replacement level. Potassium dihydrogen phosphate reacts with Pb^2+^ to form insoluble precipitates, while biochar improves cement hydration and structural integrity. Optimal performance was achieved with 40% silica fume substitution, offering a low-carbon and sustainable pathway for the resource recovery and environmental management of MSWI FA.

## 4. Thermal Treatment Technologies for Heavy Metal Recovery

Solidification/stabilization is primarily used to treat low-concentration inorganic pollutants and heavy metals, whereas thermal treatment technologies are more suitable for high-concentration organic pollutants and volatile heavy metals [70]. Through high-temperature processing, thermal treatments not only decompose organic contaminants in FA but also facilitate the formation of sintered mineral phases [21,37], thereby immobilizing heavy metals and mitigating their environmental risks [71]. Based on operational temperatures, thermal treatment approaches are typically categorized into sintering (900–1000 °C) and vitrification/melting (1000–1500 °C) [23].

### 4.1. Sintering

**Figure 6:**
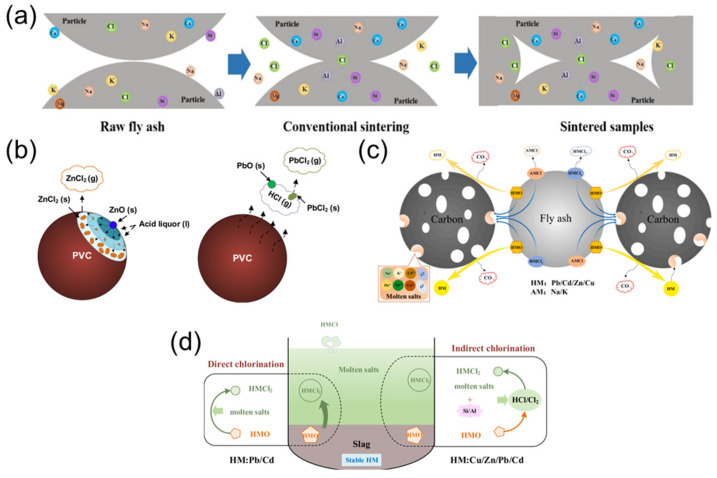
Mechanistic pathways of heavy metal transformation during thermal processes. (**a**) Conventional sintering, reproduced from [79]. 2021. Waste Manag. (**b**) Chlorination-induced migration, reproduced from [70]. 2023, Chem. Eng. J. (**c**) Co-reduction treatment, reproduced from [80]. 2020, Resour. Conserv. Recycl. (**d**) Molten salts thermal treatment, reproduced from [81]. 2020, Waste Manag.

Sintering involves the partial melting of FA particle surfaces at relatively moderate temperatures to form dense ceramic structures (Figure 6a), which immobilize heavy metals while enabling the production of high-strength ceramic pellets with significant FA incorporation [48], such as chloride salt content [72,73,74], temperature [75], and sintering duration [76], which requires optimized process conditions to control related risks. To enhance the removal efficiency of heavy metals and resource recovery from FA, a co-sintering coupled with chlorination volatilization technology was proposed [77]. By mixing hazardous waste incineration FA (HWI-FA) with MSWI FA in a 1:1 ratio and sintering at 1000 °C for 60 min, CaO from HWI-FA reacts with the Si/Al matrix of MSWI FA to generate Si/Al phases. This disrupts the dense structure of FA and promotes the chlorination volatilization of heavy metals, achieving the removal rates of 84.4% for Cu, 92.0% for Zn, 99.9% for Pb, 99.5% for Cd, and 99.7% for Cl. To suppress secondary heavy metal volatilization, an iron-based dechlorination agent-enhanced stabilization technology was also introduced [78]. Adding 2.1% iron (III) sulfate (Fe_2_(SO_4_)_3_) as a dechlorination agent reacts with chloride ions in the FA to form volatile FeCl_3_, effectively reducing the volatilization rates of Pb and Cd, while Zn and Cu volatilization remained below 3%. Through high-temperature chlorination reactions, co-sintering efficiently removes heavy metals, while the dechlorination agent enhances environmental safety by chemically blocking chlorine-mediated volatilization pathways.

### 4.2. Vitrification Techniques

Vitrification technology melts FA into a liquid state at high temperatures, forming glass-like materials upon cooling [82], which immobilizes heavy metals such as Pb and Cd within a silicate–glass network or crystalline structure [83], achieving 70–90% volume reduction [84]. The resulting product can be used as a construction aggregate for resource recovery. Studies indicate that acidic oxides in FA, such as SiO_2_, P_2_O_5_, and Al_2_O_3_, significantly increase melting temperatures, while alkaline oxides such as Na_2_O, B_2_O_3_, and CaO effectively reduce energy consumption [85,86]. A hybrid melting process has been proposed by blending MSWI FA with B2O3, reducing the 1211 °C to 986 °C with an increase in the B_2_O_3_ content from 0 to 15 wt% [87]. Wong et al. introduced a similar hybrid melting approach. By blending MSWI FA with bottom slag in a 1:5 ratio, the melting point lowered from the initial 1448 °C to 1190 °C [37].

During vitrification, heavy metals are embedded in the amorphous network structure of inorganic glass, with immobilization efficacy closely tied to the composition and structure of the FA-based material. Additionally, heavy metals can be fixed within the crystalline structure of FA-based materials by replacing existing elements or forming new crystalline phases [88]. However, stabilization effectiveness remains poor for certain volatile heavy metals [80]. To mitigate pollution and enhance resource recovery efficiency, FA recycling technology can be employed, where secondary FA is reintroduced into furnaces and converted into slag. This not only reduces contamination but also offers potential for future metal recovery [89].

Plasma vitrification technology, a promising method utilizing high-temperature plasma to convert waste such as FA into stable glassy solids, offers simplicity and ease of control [16,84]. This technology significantly enhances the immobilization of heavy metals when treating FA. It was shown that plasma vitrification reduces FA volume by 78% to 84% and effectively immobilizes heavy metals. After treatment, the leaching concentrations of Cd and Pb decreased significantly, from 1.85 mg/L to 0.01 mg/L for Cd and from 1.67 mg/L to 0.03 mg/L for Pb, respectively. However, the leaching concentration of Cr increased from 0.77 mg/L to 13.16 mg/L, indicating that high-boiling-point metals such as Cr may retain higher leachability during the melting process [16]. Similarly, research by Lin et al. observed reduced leaching concentrations for Cd decreased from 3.34 mg/L to 0.01 mg/L, and Pb from 0.97 mg/L to 0.03 mg/L, but an increase in Cr leaching under certain conditions, rising from 0.39 mg/L to 39.25 mg/L [90]. While plasma vitrification effectively reduces the environmental hazards of heavy metals, the leaching risks associated with high-boiling-point metals require further attention, necessitating process optimization or integration with complementary methods.

Thermal treatment technology processes FA at high temperatures of 1100–1300 °C, reducing its volume by approximately 67% and forming a slag structure dominated by Si-O glass networks. This encapsulates heavy metals such as chromium (Cr) and lead (Pb) within an amorphous matrix, significantly reducing leaching rates by over 90% [38]. Adding an appropriate amount of Fe_2_O_3_ (5–8 wt.%) enhances stabilization: it reacts with chromium to form FeCr_2_O_4_ spinel phases, reducing chromium leaching concentration by 98.3% at 1200 °C [91]; and interacts with PbO to generate stable PbFe_12_O_19_ phases, with dynamic leaching tests demonstrating > 98% Pb retention at 1200 °C [92]. However, excessive Fe_2_O_3_ (exceeding 10 wt.%) disrupts glass matrix continuity, compromising long-term stability. This technology achieves simultaneous FA volume reduction, detoxification, and resource utilization through crystalline phase design and structural regulation.

### 4.3. Thermal Separation Technologies

Thermal metal separation technologies involve the application of high temperatures to induce physical and chemical reactions that facilitate the separation and recovery of metals from FA. By exploiting the volatility of heavy metals under elevated temperatures, this method generates purified ash and materials enriched with metals, such as heavy metal oxides or elemental metals, thus enabling efficient metal recovery [24,70]. Common thermal separation methods include calcination at 700–1200 °C, chlorination at 700–1000 °C, reduction at 700–1000 °C, and molten salt separation at 600–800 °C [70].

Calcination separation involves heating FA to high temperatures, causing the volatilization of heavy metals or their compounds, leading to effective separation and recovery. This method is relatively simple, does not require additional reagents, and effectively reduces ash content. The efficiency of calcination is influenced by factors such as temperature and the chemical composition of FA (e.g., silica-alumina oxides and chlorides) [70]. Studies have shown that calcination temperature significantly affects the volatilization behavior of heavy metals. Specifically, for Pb and Cd, the volatilization rate increases from 30% to nearly 100% as the temperature rises from 650 °C to 900 °C. This is primarily due to the presence of Pb and Cd in the form of chlorides (e.g., PbCl_2_ and CdCl_2_) and oxides (e.g., PbO and CdO). At 650 °C, chlorides melt and facilitate volatilization, while at 900 °C, temperatures exceed the melting point of oxides, enhancing their thermal decomposition and volatilization [93,94].

Chlorination separation utilizes chlorinating agents such as gaseous HCl or solid PVC to convert metal oxides into volatile chlorides [95,96,97], thereby separating and recovering metals (Figure 6c). Under high-temperature conditions, chlorinating agents react with the metal oxides in FA, forming volatile metal chlorides that can be easily extracted. Experiments have demonstrated that at 900 °C, the volatilization rates of Pb and Zn can reach 80–100%, while those of Cu and Mn are lower (Cu < 63%, Mn~50%). The introduction of HCl significantly increases the volatilization rates of Zn, from approximately 80% to nearly 100%. However, Cu and Mn show slower volatilization, with the reaction being less efficient for these metals [98].

Reduction separation involves the use of reducing agents such as reducing gas or carbon to reduce metal oxides to their elemental forms (Figure 6d) [99,100]. The reduced metals are then easily separated and recovered. In particular, studies have shown that when FA is treated under a 10% H_2_/90% N_2_ atmosphere at 1000 °C, the volatilization rates of Zn and Cu increase significantly, while the volatilization rates of Cd, As, Sb, and Bi are around 80%. Specifically, Zn and Cu show volatilization rates of 60–90%, and Cd’s volatilization rate increases from 78% to 98% at 1000 °C, while other metals like As and Sb show volatilization rates of approximately 80% [101].

### 4.4. Molten Salt Thermal Treatment

Molten salt separation relies on the density differences between molten salts and FA to separate heavy metals [102,103]. At temperatures ranging from 600 °C to 750 °C, the migration rates of heavy metals like Cd, Cu, and Pb exceed 64%, while Zn has a relatively lower migration rate of around 32%. This method takes advantage of the interactions between heavy metals and the silica-alumina matrix, facilitating the chlorination reactions of metal oxides (e.g., ZnO, CuO) and the formation of soluble metal chlorides (e.g., ZnCl_2_, CuCl_2_). The presence of SiO_2_ in FA promotes these reactions by providing reactive chlorine sources (HCl/Cl_2_), thereby enhancing metal separation [70].

Thermal separation technologies offer various advantages depending on the specific metal recovery requirements. Calcination is a straightforward method but involves higher energy consumption; chlorination is highly efficient and operates at lower temperatures, making it more cost-effective; reduction is suitable for multi-metal systems, offering high separation efficiency; and molten salt separation provides a lower energy consumption solution, though its effectiveness for Zn is somewhat limited. Each method’s application requires a careful evaluation of the target metal characteristics and cost-effectiveness.

## 5. Leaching and Separation-Based Recovery Technologies

### 5.1. Chemical Leaching and Bioleaching

The wet extraction method involved the addition of acids to dissolve heavy metals from FA [104], which had been widely recognized as an effective and full-scale method. Among the commonly used acids, HCl and HNO_3_ were found to dissolve nearly all metal elements present in FA [105,106]. In contrast, sulfuric acid (H_2_SO_4_) was less effective for certain metals, as it formed insoluble precipitates with Ca^2+^ and Pb^2+^, thereby limiting their extraction efficiency [107,108]. Although sulfuric acid leaching achieved extraction efficiencies above 60% for metals such as Mg, Zn, and Al, its use significantly increased treatment costs. To address this limitation, researchers proposed combining chemical leaching with biological leaching as a more sustainable and cost-effective strategy for enhanced heavy metal recovery from FA. However, the biological leaching process has only been demonstrated in a 2 L laboratory-scale reactor, indicating that significant advancements are still required before it can be feasibly scaled up for full-scale applications [109].

### 5.2. Electrochemical Separation and Enrichment

Electrochemical techniques have emerged as promising strategies for the recovery and selective separation of heavy metals from FA. By leveraging redox potential gradients, these methods facilitate the targeted migration and deposition of metal ions, thereby enabling efficient separation and recovery. Among the various electrochemical approaches, electrodialysis (ED) and electrokinetic remediation (EKR) are the most commonly applied [110].

**Figure 7:**
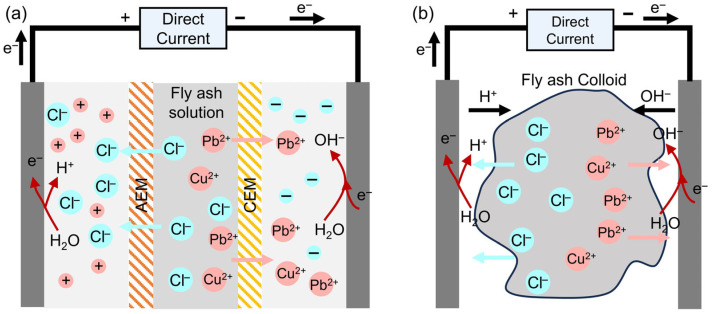
Mechanism of (**a**) electrodialysis and (**b**) electrokinetic remediation.

Electrodialysis (ED), as a typical electrochemical technology, enables selective migration of heavy metals through ion exchange membranes [111]. This technology combines environmental friendliness, high efficiency, and continuous operation, offering a novel approach for heavy metal recovery. An ED system typically consists of an anode chamber, a cathode chamber, and a central chamber, separated by ion exchange membranes [112], as illustrated in Figure 7a. When an electric field is applied, oxidation-reduction reactions occur at the anode and cathode, generating H^+^ and OH^−^ ions, respectively. Ions in the central chamber migrate toward the electrode chambers under the electric field, with positively charged heavy metal ions such as Cu^2+^ and Zn^2+^ crossing the anion exchange membrane to reach the cathode chamber for recovery.

Electrokinetic remediation (EKR) removes heavy metals by inducing their directional migration under a direct current electric field. A typical EKR setup comprises a sample chamber flanked by electrode compartments, where a graphite anode and a stainless steel cathode are used to establish the electric field [113]. As illustrated in Figure 7b, this process facilitates metal ion transport, regulates dissolution-precipitation equilibria via electrolytic reactions, and enables the removal of heavy metals from FA through enrichment and separation in the electrode zones.

Electrochemical recovery technologies enable the extraction of metals and valuable elements from waste, particularly heavy metals such as Cu, Pb, and Zn [114,115,116]. These recovered metals can be converted into pure forms through electrolysis for use in electronic products, batteries, and construction materials. Additionally, elements like phosphorus and potassium from fly ash can be recovered for use in agricultural fertilizers. Resource recovery not only helps reduce environmental pollution but also supports the development of a circular economy by reducing dependence on raw materials [117], lowering energy consumption, and minimizing resource extraction, with electrochemical technologies playing a vital role in promoting sustainable resource management [118].

## 6. Conclusions

Municipal solid waste incineration FA is enriched with various heavy metals in complex chemical forms, exhibiting significant environmental persistence and potential health risks due to their mobility, leachability, and bioavailability. A thorough understanding of the occurrence, speciation, and transformation behaviors of heavy metals is fundamental to accurately assessing ecological risks and selecting appropriate remediation technologies.

Solidification/stabilization technologies, particularly those employing cementitious materials or chemical amendments, remain widely adopted due to their operational simplicity and short-term effectiveness in immobilizing metals. However, concerns persist regarding long-term stability, material expansion, and limited potential for resource recovery. Thermal treatment approaches-such as sintering, vitrification, and thermal separation-offer advantages in volume reduction and heavy metal detoxification. Recent advancements, including co-sintering with additives, plasma vitrification, and molten salt-assisted separation, have improved treatment efficiency and product stability, although high energy demands and operational costs remain key barriers. Alternatively, chemical leaching, bioleaching, and electrochemical methods are emerging as viable solutions for the selective recovery of heavy metals, contributing to the advancement of circular economy goals. Nonetheless, issues such as secondary pollution, reagent consumption, and the management of complex residues warrant further investigation. Meanwhile, due to the varying levels of technological maturity and application scales, a standardized and quantitative comparison of treatment methods in terms of efficiency, leachability, scalability, environmental impact, and cost remains difficult at this stage.

To better address the current challenges such as long-term instability, low resource recovery efficiency, high energy consumption, and poor scalability, future research should focus on developing integrated, low-carbon, and resource-oriented treatment systems for MSWI fly ash. This includes advancing hybrid processes that couple stabilization with downstream resource recovery, clarifying the transformation mechanisms and mobility of heavy metals under realistic disposal or reuse conditions, and optimizing energy-efficient thermochemical treatments such as low-temperature sintering and partial vitrification. In addition, the development of intelligent risk assessment and decision-support tools based on life-cycle thinking and machine learning is essential. Promoting environmentally friendly technologies, including recyclable chelating agents and bio-electrochemical recovery systems, will help minimize secondary pollution. Finally, techno-economic evaluations and effective policy integration are crucial to support large-scale implementation and to facilitate the transition from conventional pollution control to a circular resource utilization paradigm.

## Data Availability

The data supporting the findings of this study are included within the paper and Supplementary Materials and are available from the corresponding authors on request.

## Supplementary Materials

The following supporting information can be downloaded at: https://www.mdpi.com/article/10.3390/toxics13080695/s1. Table S1: Changes in municipal solid waste (MSW) and distribution of treatment capacity proportions in China from 2014 to 2023; Table S2: Changes in yield of municipal solid waste (MSW, 10^4^ tons) in each province in 2014, 2017, 2020, and 2023, respectively.
