# Supplementary material for: Recent Advances in Heavy Metal Stabilization and Resource Recovery from Municipal Solid Waste Incineration Fly Ash
Source: Toxics. 2025 Aug 20;13(8):695. doi: 10.3390/toxics13080695 (PMC12390417; doi:10.3390/toxics13080695)
Supplement: Supplementary file 1 [file toxics-13-00695-s001.zip › toxics-3724061-supplementary.pdf]

Supporting information

Recent advances in heavy metal stabilization and resource recovery from municipal solid waste incineration fly ash

Yunfei He[1,2,3], Yue Jiang[1,2], Lingwei Ren[4], Chenyiyi Qian[1], Han Zhang[1], Yuchi Zhong[4], Xuetong Qu[1,2], Jibo Dou[1,2], Shuai Zhang[1,2], Jiafeng Ding[1,2*], Hangjun Zhang[1,2]

[1] School of Engineering, Hangzhou Normal University, 310018, Hangzhou, Zhejiang, China

[2] Zhejiang Provincial Key Laboratory of Wetland Intelligent Monitoring and Ecological Restoration, 311121, Hangzhou, Zhejiang, China

[3] School of the Environment, Nanjing University, Nanjing, 210023, PR China

[4] Hangzhou Fuyang Huilong environmental protection Technology Co., LTD, 330183, Hangzhou, Zhejiang, China

***Corresponding authors.**

Jiafeng Ding: djf101@126.com (Email), +86-571-28861265 (Tel)

Table S1. Changes in municipal solid waste (MSW) and distribution of treatment capacity proportions in China from 2014 to 2023.

**Year**	2014	2015	2016	2017	2018	2019	2020	2021	2022	2023
The proportion of incineration capacity (%)	35	38	41	44	48	53	59	68	73	75
The proportion of landfill capacity (%)	63	60	56	53	49	42	35	25	19	15
The proportion of other capacity (%)	2	2	2	3	4	5	6	7	8	10
MSW landfill volume (10^4 tons)	10744.3	11483.1	11866.4	12037.6	11706	10948	7771.5	5208.5	3043.2	1892.6
MSW incineration volume (10^4 tons)	5329.9	6175.5	7378.4	8463.3	10184.9	12174.2	14607.6	18019.7	19502.1	20954.4
The yield of MSW (10^4 tons)	17860.2	19141.9	19141.9	19141.9	19141.9	19141.9	19141.9	19141.9	19141.9	19141.9

Note: Date was collected from National bureau of statistics of China

Table S2. Changes in yield of municipal solid waste (MSW, 10^4 tons) in each province in 2014, 2017, 2020, and 2023, respectively.

**Year**	2014	2017	2020	2023
Beijing	730.8	923.7	797.5	758.8
Tianjin	208.7	289.8	306.5	301.9
Hebei	531.9	698.0	786.2	784.4
Shanxi	409.7	454.5	460.7	518.5
Neimenggu	311.8	367.0	387.4	356.0
Liaoning	840.0	856.3	988.6	1029.3
Jilin	312.4	355.3	464.2	452.6
Heilongjiang	325.7	457.7	497.0	523.8
Shanghai	608.4	743.1	868.1	974.8
Jiangsu	1326.9	1734.7	1870.5	2081.7
Zhejiang	1229.0	1454.6	1444.9	1467.8
Anhui	462.5	611.9	660.7	771.9
Fujian	586.1	781.5	878.5	878.9
Jiangxi	287.1	440.5	527.5	553.3
Shandong	958.5	1591.3	1673.9	1804.5
Henan	773.1	982.1	1129.5	1121.1
Hubei	666.6	907.0	987.4	1085.8
Hunan	599.0	763.0	797.1	904
Guangdong	1912.7	2591.2	3101.0	3388.8
Guangxi	323.3	438.0	519.6	615.3
Hainan	144.0	213.0	253.6	317.4
Chongqing	396.2	526.7	589.7	643.3
Sichuan	743.9	975.4	1136.5	1321.7

Guizhou	255.3	308.1	350.8	461.9
Yunnan	323.2	379.4	487.4	544.9
Xizang	ND	44.4	62.1	70.8
Shaanxi	496.1	375.3	549.6	715.9
Gansu	158.4	250.5	272.6	282.9
Qinghai	67.0	73.7	115.4	114.8
Ningxia	110.4	117.9	126.8	122.8
Xinjiang	295.3	328.9	360.8	432.1
Taiwan	ND	ND	ND	ND

Note: Date was collected from National bureau of statistics of China, ND represents the data was not collected.
